# Simultaneous Quantification of 11 Phenolic Compounds and Consistency Evaluation in Four *Dendrobium* Species Used as Ingredients of the Traditional Chinese Medicine Shihu

**DOI:** 10.3389/fnut.2021.771078

**Published:** 2021-11-04

**Authors:** An-Ling Zhu, Jing-Wen Hao, Lei Liu, Qi Wang, Nai-Dong Chen, Guang-Lin Wang, Xiao-Quan Liu, Qiang Li, Hui-Min Xu, Wei-Han Yang

**Affiliations:** ^1^School of Pharmacy, Anhui University of Traditional Chinese Medicine, Hefei, China; ^2^College of Biotechnology and Pharmaceutical Engineering, West Anhui University, Lu'an, China; ^3^Anhui Engineering Laboratory for Conservation and Sustainable Utilization of Traditional Chinese Medicine Resource, Lu'an, China; ^4^Medical College, Bengbu Medical College, Bengbu, China; ^5^Endocrinology Department, Lu'an Hospital of Anhui Medical University, Lu'an, China

**Keywords:** *Dendrobium*, Shihu, one medicine from multiple origins, polyphenol, consistency evaluation

## Abstract

The interchangeable use of different herbs to prepare the same formulation is a common practice in Traditional Chinese Medicine (TCM). However, this practice would require the component herbs to share similar compositions, at least in terms of the bioactive agents, to ensure they can replace each other in drug preparation. In this study, we developed an effective and comprehensive high-performance liquid chromatography-diode array detector (HPLC-DAD) method for simultaneous analysis of 11 phenolic compounds in the methanol extracts of *Dendrobium huoshanense, Dendrobium nobile* (*D. nobile*), *Dendrobium chrysotoxum* (*D. chrysotoxum*), and *Dendrobium fimbriatum* (*D. fimbriatum*), which have been identified as interchangeable ingredients for the same TCM preparation “Shihu” in the Chinese pharmacopeia (ChP). The consistency of the four *Dendrobium* species was evaluated on the basis of the presence of the 11 investigated compounds and the HPLC fingerprints of the methanol extracts of the plants. When gradient elution was performed with a solvent system of acetonitrile and water on a Zorbax Eclipse XDB-C18 (150 mm × 4.6 mm, 5 μm) with monitoring at 220 nm, all 11 investigated compounds were isolated at the baseline. The established HPLC method showed excellent linearity (all analytical curves showed relative coefficients [R^2^] > 0.999), sensitivity, precision (relative standard deviation [RSD] < 2%), and accuracy (recovery, 90.65–99.17%). These findings confirmed that the method we constructed was reliable. Quantification analysis showed significant differences in the contents of the investigated polyphenols in the four *Dendrobium* species. Evaluations of consistency revealed that the similarities among the four species were 0.299–0.906 in assessments based on the 11 polyphenols and 0.685–0.968 in assessments based on HPLC fingerprints. Thus, the components of the four *Dendrobium* species may be significantly different, and more experiments are required to determine whether they can be used interchangeably in the same amounts for preparing the formulation according to ChP.

## Introduction

*Dendrobium* is one of the largest groups of the family Orchidaceae (1–3), with more than 1,100 species worldwide and 74 species and two varieties in China (4). Most *Dendrobium* plants have long been used as ingredients of the famous Traditional Chinese Medicine (TCM) Shihu in China. Among them, *Dendrobium huoshanense* C. Z. Tang et S. J. Cheng*, Dendrobium nobile* Lindl*, Dendrobium chrysotoxum* Lindl, and *Dendrobium fimbriatum* Hook are the most important ingredients of Shihu. These four *Dendrobium* species are believed to perform the same functions and thus can be interchangeably used for the preparation of Shihu according to the Chinese pharmacopeia (ChP). According to TCM theory, plants belonging to all the four *Dendrobium* species have a sweet and slightly cold nature, and their main functions are to nourish yin, clear away heat-evil, tonify the stomach, and promote fluid (5, 6). They are mainly used for treating febrile injuries, insufficiencies of stomach yin, lack of appetite and impotence, and other similar conditions. The previous phytochemical studies showed that Shihu is rich in polysaccharides (7–9), polyphenols (10, 11), and alkaloids (12–14). As one of the main active components of Shihu, polyphenols can improve the immune function of the body and show anti-cataract, anti-aging, anti-mutation, anti-tumor (15, 16), anti-inflammatory (17), and other pharmacological activities.

The interchangeable use of multiple ingredients to prepare the same formulation is a common practice in TCM preparations other than Shihu. However, the chemical ingredients of medicinal plants are the basis of their pharmaceutical activities, and the accumulation of bioactive components can be greatly affected by hereditary characteristics and growth environments. Thus, even the components of the same plant can be expected to vary largely with changes in habitats, and even larger differences in chemical compositions may appear in members of different species. Therefore, the rationality of the interchangeable use of the four *Dendrobium* species needs to be validated.

In this paper, we aimed to address this issue by developing an accurate and systematic high-performance liquid chromatography-diode array detector (HPLC-DAD) method for simultaneous analysis of 11 phenolic compounds in *Dendrobium huoshanense* (*D. huoshanense*)*, Dendrobium nobile* (*D. nobile*), *D. chrysotoxum* (*D. chrysotoxum*), and *Dendrobium fimbriatum* (*D. fimbriatum*). The consistency of the four *Dendrobium* plants was then evaluated on the basis of the 11 investigated polyphenols and the HPLC fingerprints of the methanol extract to clarify the rationality of interchangeably using these four *Dendrobium* species to prepare the same medicine and, thus, provide a reference method for evaluation of other multiple-origin TCM preparations.

## Materials and Methods

### Chemicals and Reagents

**Figure 1:**
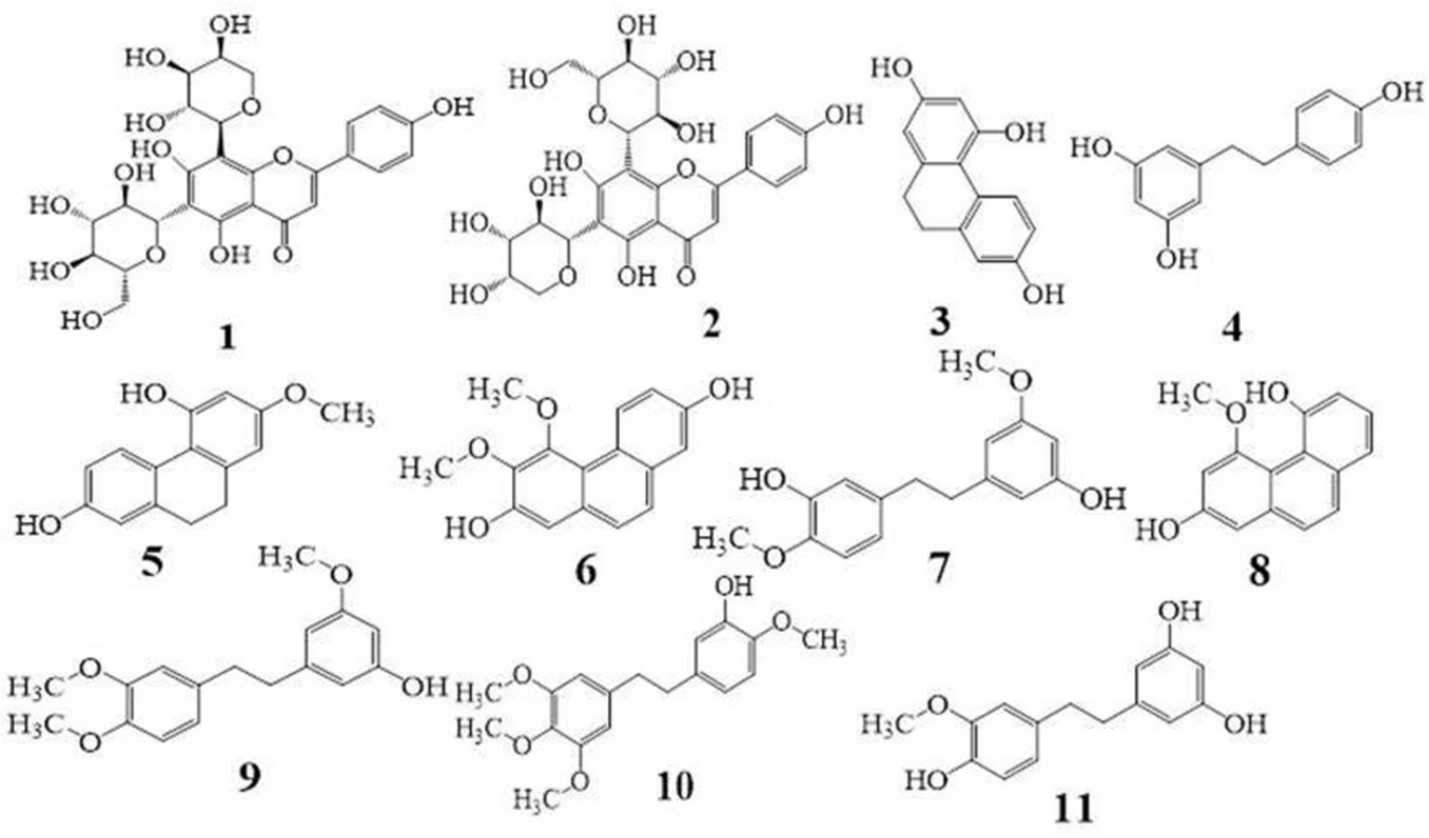
Chemical structures of the 11 phenolic compounds. **(1)** schaftoside; **(2)** isoschaftoside; **(3)** 2,4,7-trihydroxy-9,10-dihydrophenanthrene; **(4)** dihydroresveratrol; **(5)** coelonin; **(6)** nudol; **(7)** gigantol; **(8)** 4-methoxy-2,5-phenanthrenediol; **(9)** 3-hydroxy-3,4,5-trimethoxybibenzy; **(10)** erianin; and **(11)** Tristin.

Chromatographic-grade solvents, namely, methanol and acetonitrile, were obtained from Tedia High-purity Solvent Co., Ltd. (Fairfield, OH, USA), and formic acid was purchased from Aladdin Reagent Co., Ltd. (Shanghai, China) The double-distilled water was prepared in the laboratory by using a Milli-Q system (Millipore, Bedford, MA, USA). Schaftoside (1), isoschaftoside (2), 2,4,7-trihydroxy-9,10-dihydrophenanthrene (3), dihydroresveratrol (4), coelonin (5), nudol (6), gigantol (7), 4-methoxy-2,5- phenanthrenediol (8), 3-hydroxy-3,4,5-trimethoxybibenzy (9), erianin (10), and tristin (11) were purchased from Sichuan Weikeqi Biological Technology Co., Ltd. (Sichuan province, Chengdu, China). The purity of all the reference standards was above 98%. The structures of these standards are shown in Figure 1.

### Sample Processing and Solid-Phase Extraction

*Dendrobium huoshanense* was collected on November 14, 2020, from Huoshan County, Anhui Province, China. *D. nobile, D. chrysotoxum*, and *D. fimbriatum* were collected on November 14, 2020, from Wenshan, Yunnan Province, China. All the plants were identified by Prof. Nai-Fu Chen, West Anhui University, China. Six replicates of each Dendrobium species were collected and analyzed. The samples were prepared as follows: the fresh stems were freeze-dried to constant weight at −50°C, separately powdered in a blender, and screened using a 200-mesh sieve. The *Dendrobium* powder was weighed (2.0 g) and treated by condensation reflux three times by using 120 ml of 90% methanol at 80°C, with each treatment lasting for 1 h. The methanol extracts of the four *Dendrobium* species were obtained by decompressing and concentrating the filtrate to dryness.

Solid-phase extraction was performed using a CNWBOND LC-C18 solid-phase extraction (SPE) cartridge (2.0 g/10 ml; ANPEL Laboratory Technologies Inc., Shanghai, China) as follows: the cartridge was activated using 50 ml of methanol followed by 25 ml of Milli-Q water. Next, 100 mg of methanol extract was precisely weighed, dissolved in 20 ml of distilled water, and filtered through a 0.22-μm microporous membrane. The sample solution was then injected into the activated SPE cartridge. The solid-phase extraction procedure was performed as follows: first, elution was performed with 45 ml of Milli-Q water, followed by elution with 45 ml of 20% methanol solution, and a final elution with 60 ml of 60% methanol solution. The eluate obtained after the 60% methanol elution was decompressed and concentrated to dryness for further analyses.

### Preparation of the Working Solution

Standard stock solutions of the reference standards were prepared by mixing and dissolving appropriate amounts of the pure substances in methanol. The initial concentrations of compounds 1–11 were 1.38, 1.55, 1.0, 1.05, 1.23, 1.0, 2, 1.1, 1.0, 1.55, and 1.04 mg/ml, respectively. Then, the mixed standard solution was gradient-diluted to obtain the working solutions. All the solutions were stored in a refrigerator at 4°C.

### Instrument and Chromatographic Conditions

**Figure 2:**
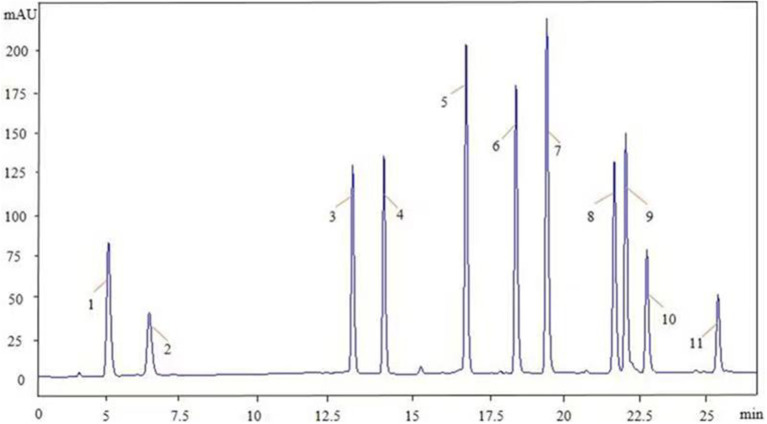
The HPLC chromatogram of the 11 mixed standards. The peak numbers 1–11 represent the standard compounds 1–11 in Figure 1. HPLC, high-performance liquid chromatography.

Chromatographic analyses were performed using the Agilent1260 HPLC system (Santa Clara, CA, USA) equipped with a quaternary pump (G1311B), automatic injector (G7129A), a column temperature controller, and a diode array detector (G4212B), all controlled by an Agilent ChemStation. Chromatographic separation was performed on a Zorbax Eclipse XDB-C18 (150 × 4.6 mm, 5 μm) at a column temperature of 35°C. The mobile phase was composed of acetonitrile (A) and Milli-Q water (B) with the following gradient program: 0–6 min, 14.7–16.2% A; 6–25 min, 16.2–60% A; 25–27 min, 60–100% A; 27–30 min, 100% A; 30–32 min, 100–14.7% A; and 32–35 min, 14.7% A. The flow rate was 1.0 ml/min, and the injection volume was 10.0 μl. The DAD spectra were acquired over a scan range of 200–400 nm. Subsequently, HPLC chromatography of the 11 mixed standards was performed (Figure 2).

### Validation of the HPLC Method

#### Calibration Curves

Under optimized analytical conditions, calibration curves were constructed by plotting the peak area (x) as a function of the corresponding mass (y, ng) of the working solution with a minimum of eight calibration concentration levels. The limits of detection (LOD) and quantification (LOQ) were measured in relation to signal-to-noise ratios (S/N) of 3 and 10, respectively.

#### Precision, Repeatability, Stability, and Recovery

A mixed standard solution with known intermediate concentrations was continuously injected six times according to the chromatographic conditions described in Instrument and Chromatographic Conditions, the peak area was determined, and the relative standard deviation (RSD) was calculated as a measure of precision. Repeatability was evaluated by determining the RSDs for the peak areas of the standards from six replicate analyses of the working solution with known intermediate concentrations, and the stability of the 11 compounds was investigated by using a single standard solution sampled at 0, 2, 4, 8, 12, and 24 h in accordance with the chromatographic conditions under Instrument and Chromatographic Conditions.

To evaluate the accuracy of the method, three different concentration levels (75, 100, and 125%) of the standard solutions were added to samples with known content for recovery tests. Three parallel samples were extracted for each level. The rate of recovery was calculated with the amount detected by comparing the amount added.

## Results and Discussion

### Qualitative Analysis

In this paper, compounds 1–11 in the *Dendrobium* samples were identified by the comparison of the HPLC retention times (RTs), UV spectra, and mass spectrometry between the standards and the possible polyphenol peaks.

As shown in Table 1, in our established HPLC chromatogram conditions, the RTs of the possible polyphenol peaks in the HPLC chromatograms of the four investigated *Dendrobium* species were very close to those of the standards for compounds 1–11, and the UV spectra of the possible polyphenol peaks (all showing peak purities above 94%) were very similar to those of the standards. Subsequently, schaftoside (1), isoschaftoside (2), 2,4,7-trihydroxy-9,10-dihydrophenanthrene (3), dihydroresveratrol (4), nudol (6), gigantol (7), and tristin (11) were identified from *D. huoshanense*; schaftoside (1), isoschaftoside (2), 2,4,7-trihydroxy-9,10-dihydro-phenanthrene, dihydroresveratrol (4), nudol (6), erianin (10), and tristin (11) were identified from *D. nobile*; schaftoside (1), isoschaftoside (2), 2,4,7-trihydroxy-9,10-dihydrophenan-hrene (3), dihydroresveratrol (4), nudol (6), and tristin (11) were identified from *D. chrysotoxum*; and schaftoside (1), isoschaftoside (2), 2,4,7-trihydroxy-9,10-dihydrophenanthrene (3), dihydroresveratrol (4), coelonin (5), and tristin (11) were identified from *D. fimbriatum*. Thus, the 11 polyphenols were not uniformly distributed in the four *Dendrobium* species used for preparing Shihu.

**Table 1:** The RT and the UV spectra of the standards and the possible polyphenols obtained by HPLC-DAD analysis.

	**Standards**		**Possible polyphenol peaks in the samples**		
**Compound**	**RT (min)**	**UV spectrum**	**RT (min)**	**UV spectrum**	**Purity (100%)**
1	5.11–5.12	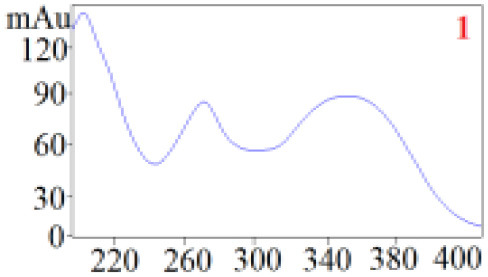	5.07–5.32	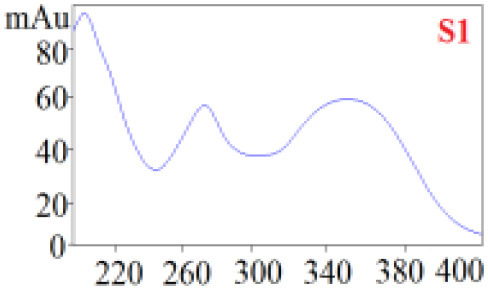	99.24
2	6.45–6.46	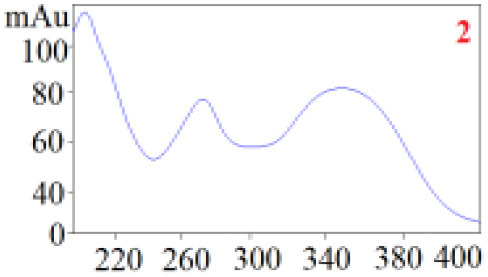	6.36–6.58	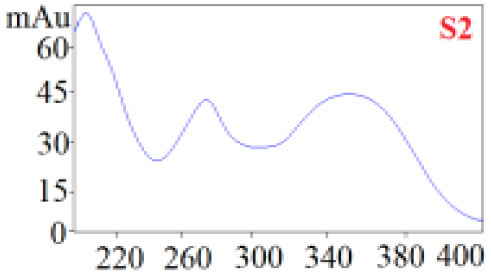	99.48
3	13.08–13.09	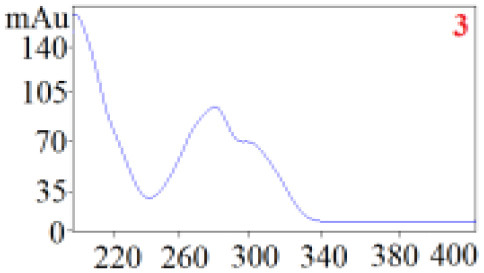	13.01–13.17	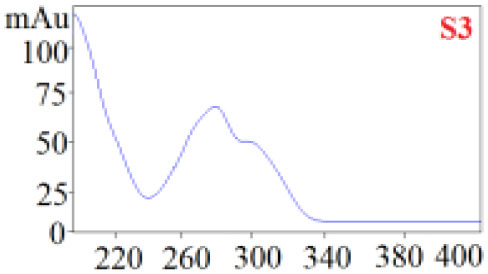	99.99
4	14.09–14.10	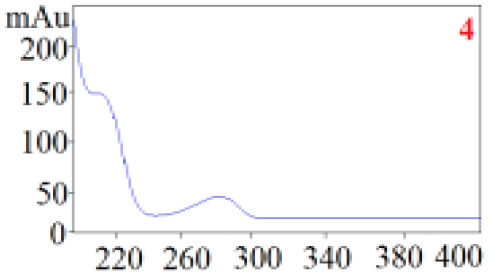	14.09–14.20	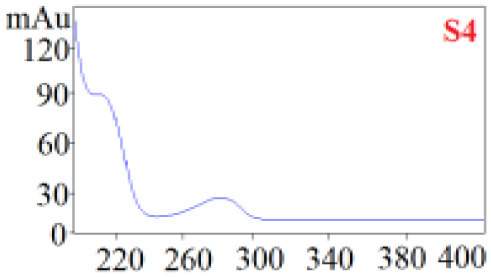	99.99
5	16.68–16.78	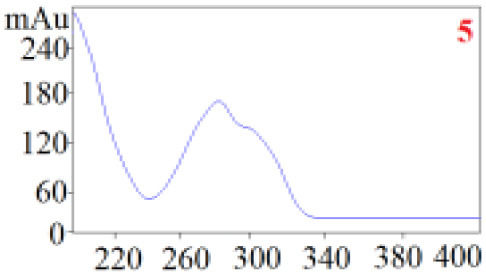	16.87–16.94	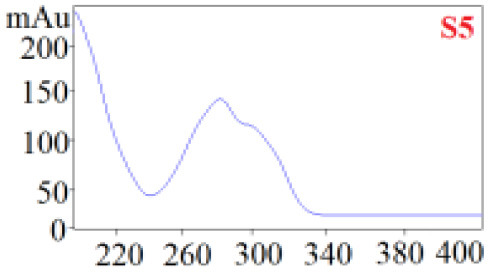	96.40
6	18.41–18.45	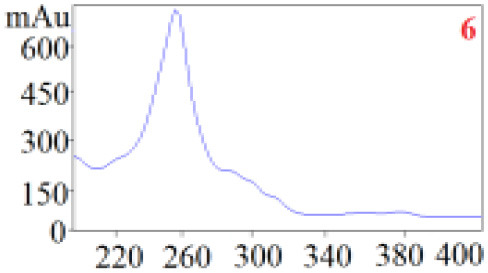	18.31–18.57	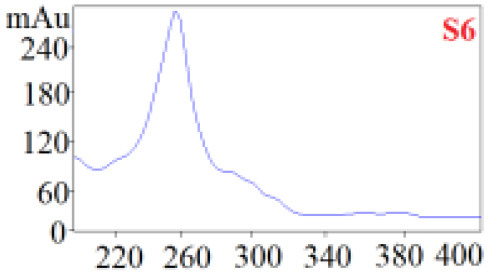	99.69
7	19.33–19.41	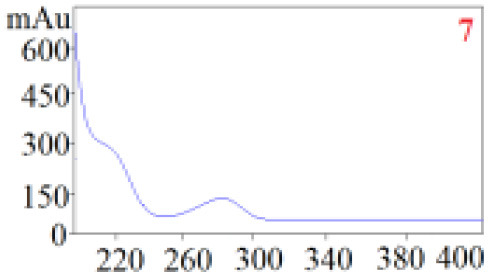	19.35–19.59	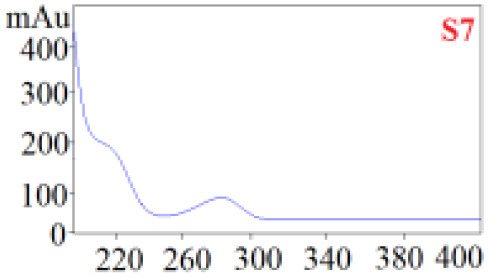	99.86
8	21.62–21.67	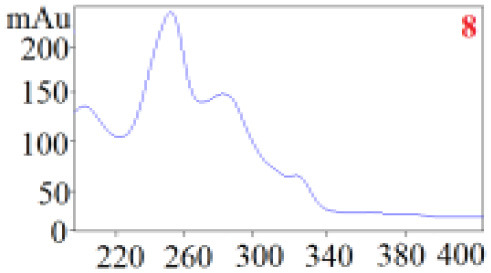	21.62–21.76	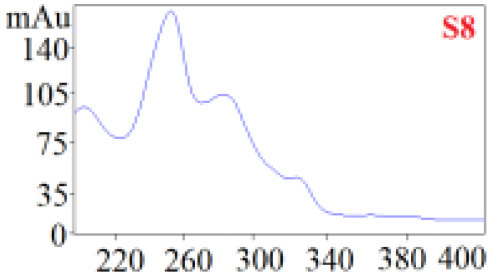	–
9	21.89–21.99	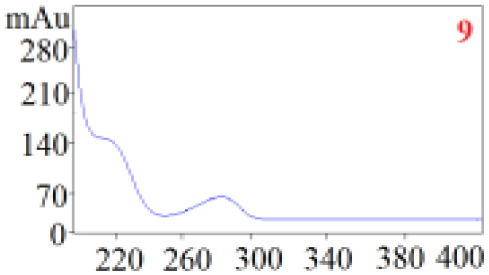	21.63–21.76	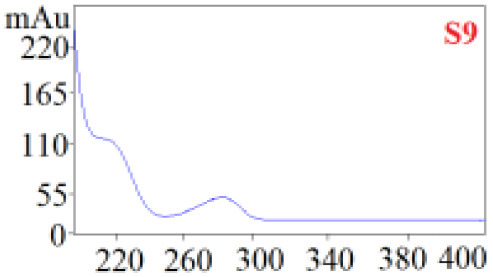	–
10	22.68–22.69	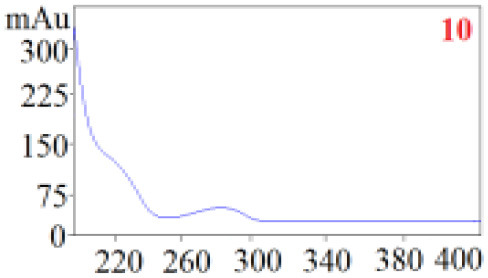	22.67–22.87	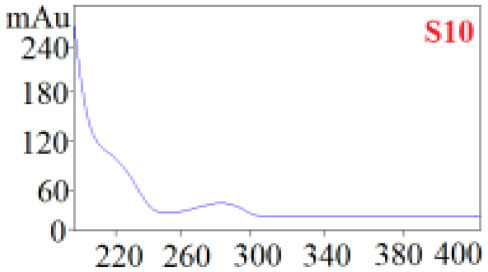	99.99
11	25.00–25.01	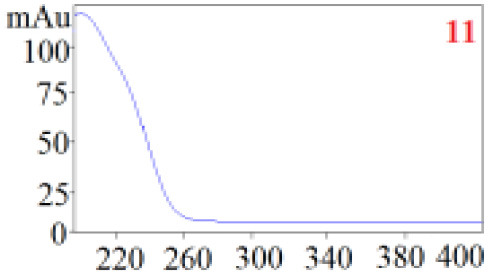	24.95–25.27	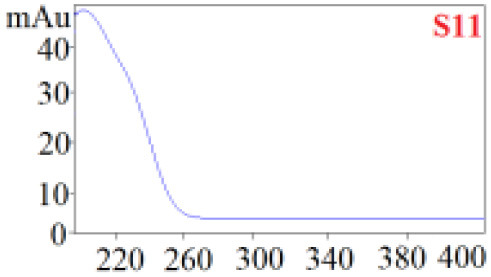	99.94

*The numbers 1–11 represent the standard compounds 1–11 in Figure 1*.

*RTs, retention times; HPLC-DAD, high-performance liquid chromatography-diode array detector*.

In our study, samples of four different *Dendrobium* species were analyzed by the established Ultra High Performance Liquid Chromatography Quadrupole-Time of Flight Mass Spectrometry/Mass Spectrometry (UPLC-Q-TOF-MS/MS) system. The target compounds in samples were identified by comparing the observed MS and MS/MS spectrum with those of standards. In our experiments, a total of nine polyphenols were detected and identified from the four forms of four different original plants of Shihu by comparing their accurate mass and fragmentation pattern with their corresponding standard compounds.

As it was shown in Tables 2, 3, in our established UPLC-Q-TOF-MS/MS conditions, compound schaftoside (1), isoschaftoside (2), 2,4,7-trihydroxy-9,10-dihydrophenanthrene (3), dihydroresveratrol (4), coelonin (5), nudol (6), gigantol (7), erianin (10), and tristin (11) were identified in the four Dendrobium plants. Nine polyphenols in the four Dendrobium plants demonstrated quasi-molecular ions [M + H] ^+^ in positive ion mode. Compounds 1 and 2 exhibited the same molecular ion at m/z 565.1552 [M + H] ^+^. It is suggested that these two components may be isomers. Their fragment ions were different, where compound 1 MS2 ions at 177.0180([M + H] ^+^), 121.0281 ([M + H] ^+^), 191.0326 ([M + H] ^+^), compound 2 MS^2^ ions at 122.0281([M + H] ^+^), 177.0193 ([M + H] ^+^), 189.0879([M + H] ^+^).

**Table 2:** The MS spectrum of the standards and the possible polyphenols obtained by Q-TOF-MS analysis.

**Standards**		**Possible polyphenol in the samples**
**Compound**	**MS**	**MS**
1	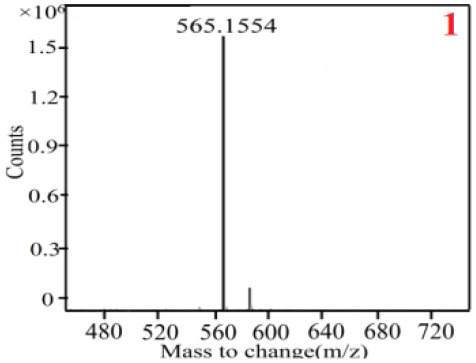	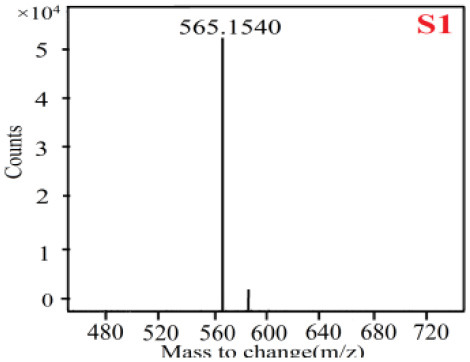
2	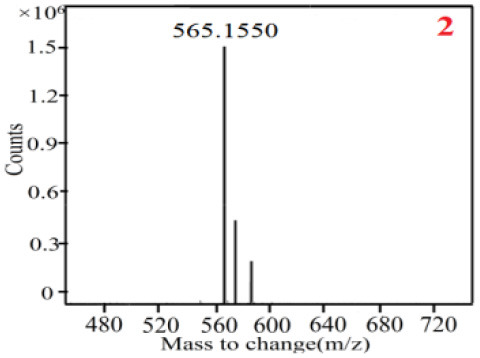	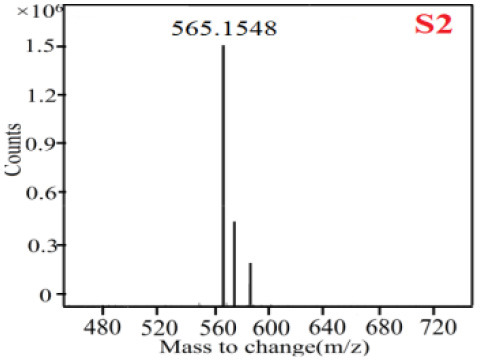
3	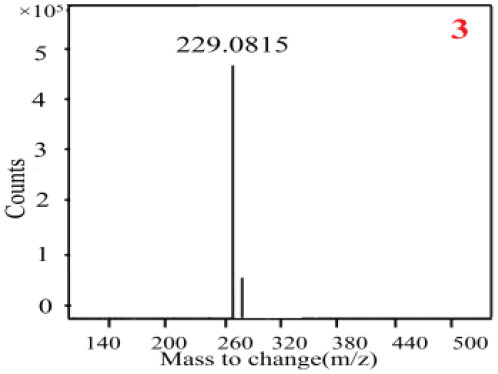	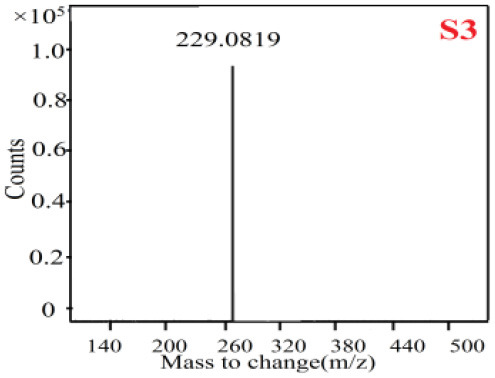
4	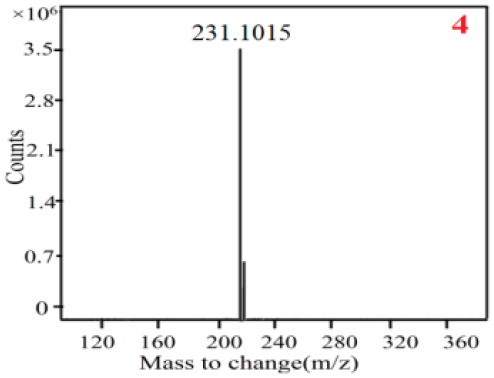	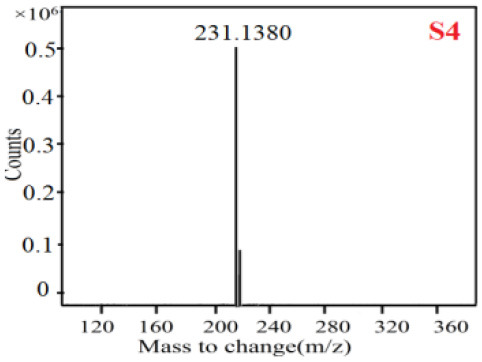
5	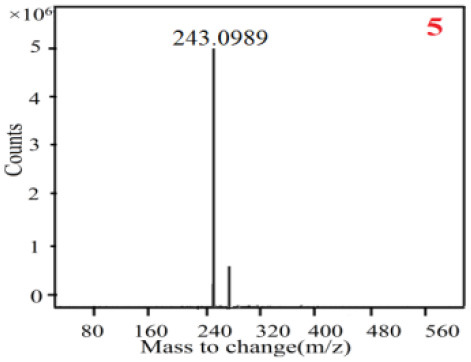	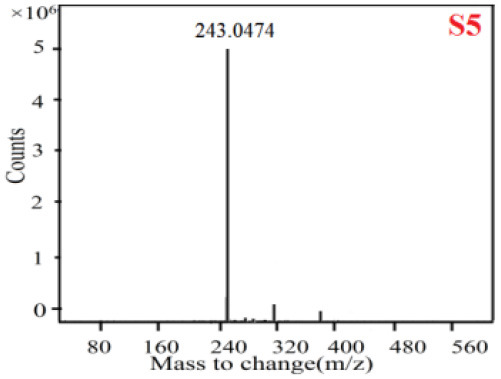
6	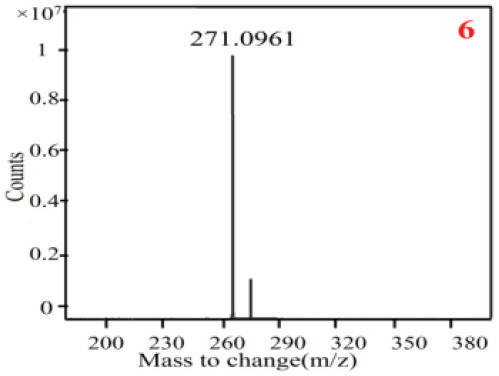	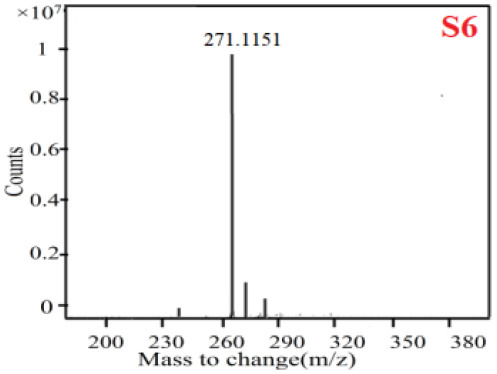
7	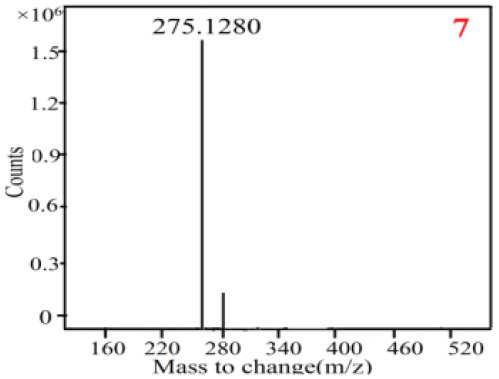	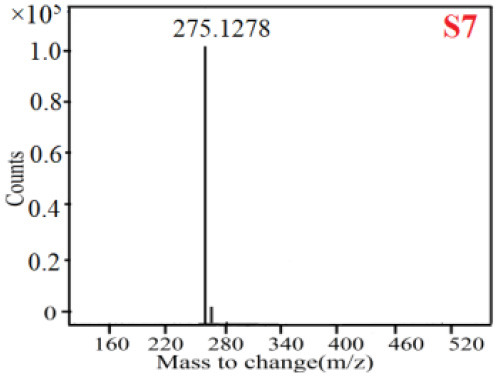
8	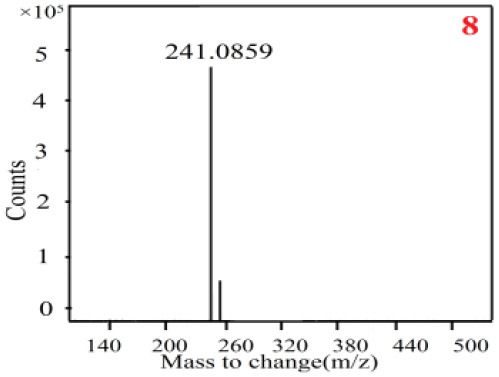	
9	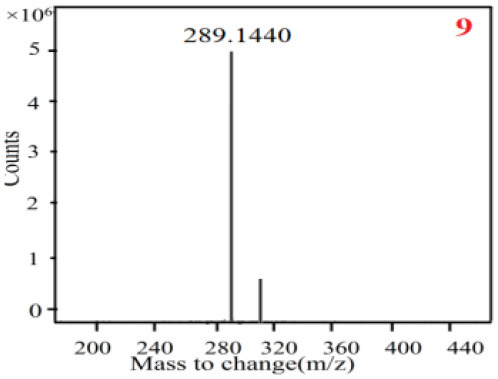	
10	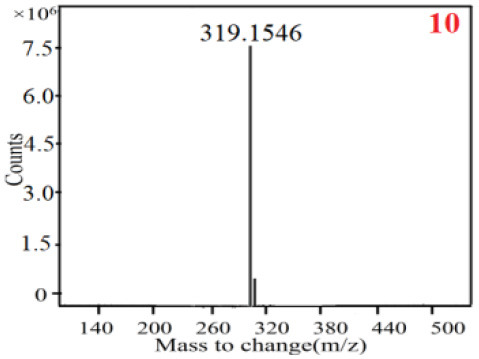	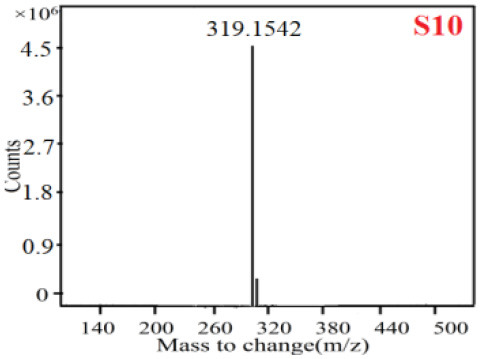
11	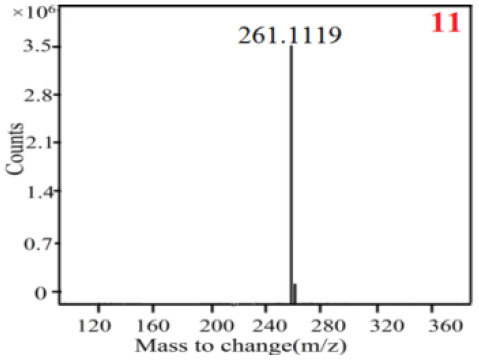	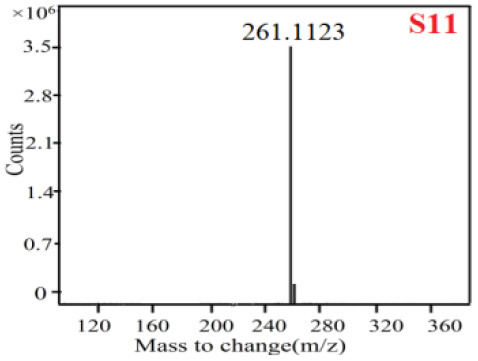

*The numbers 1–11 represent the standard compounds 1–11 in Figure 1*.

**Table 3:** The MS/MS spectrum of the standards and the possible polyphenols obtained by Q-TOF-MS analysis.

**Standards**		**Possible polyphenol in the samples**
**Compound**	**MS**	**MS**
1	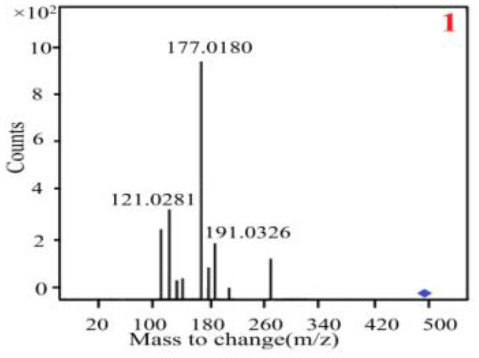	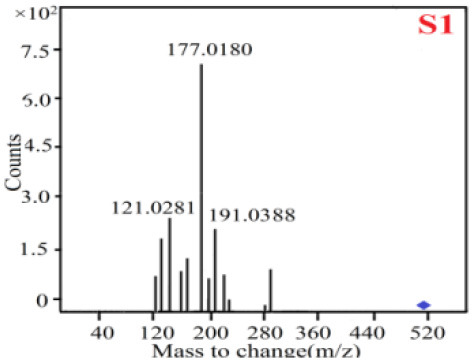
2	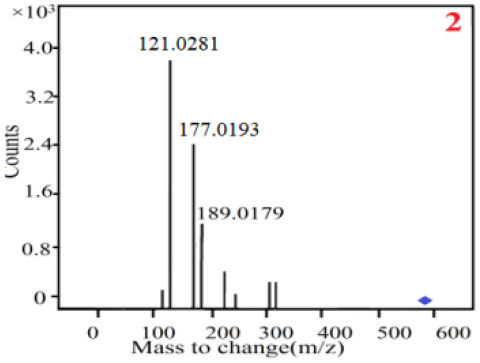	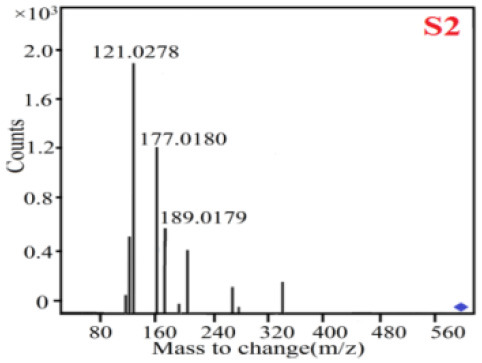
3	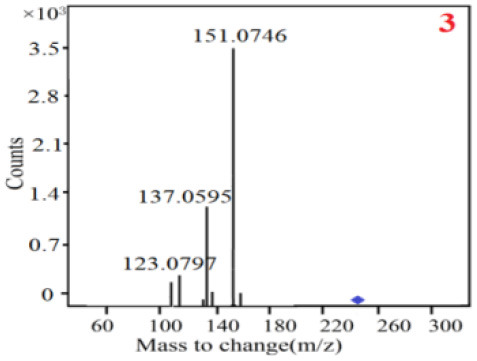	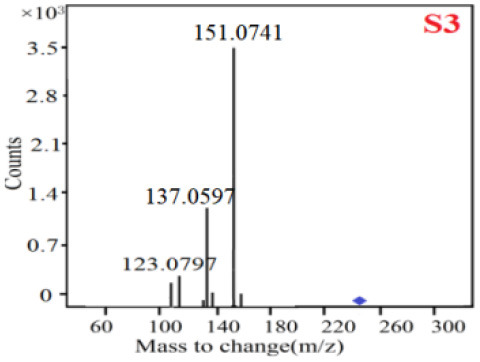
4	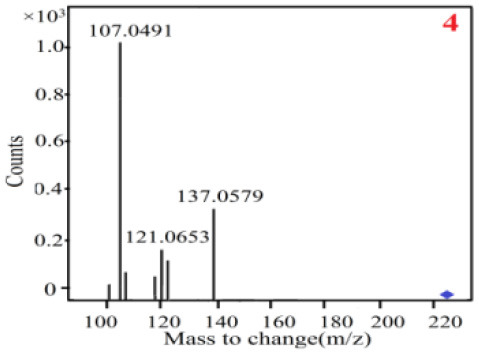	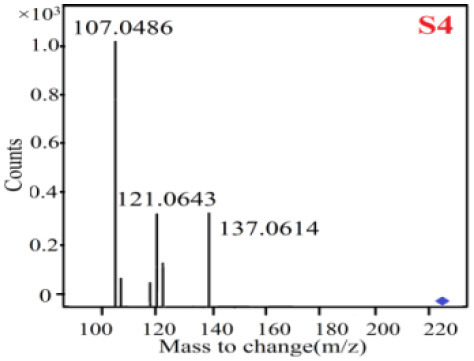
5	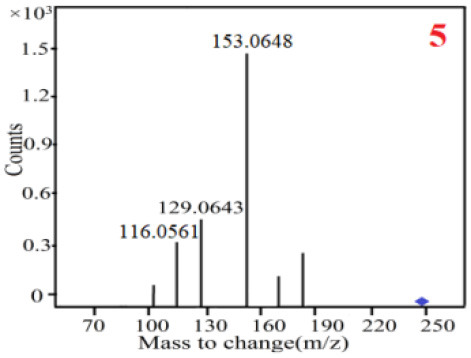	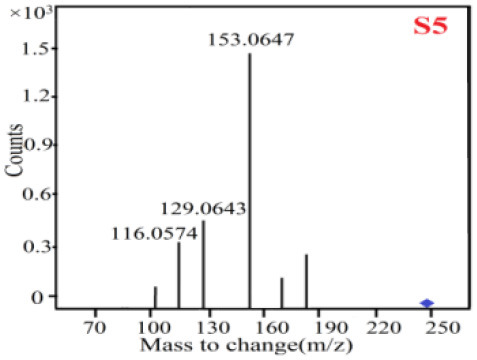
6	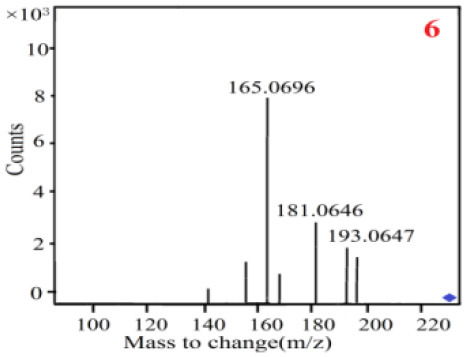	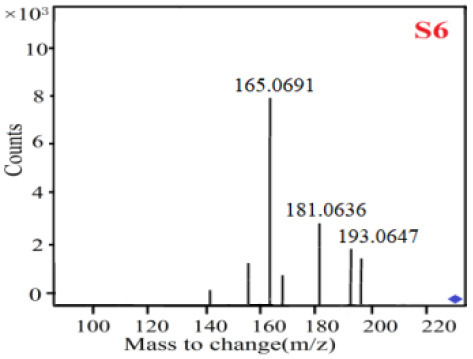
7	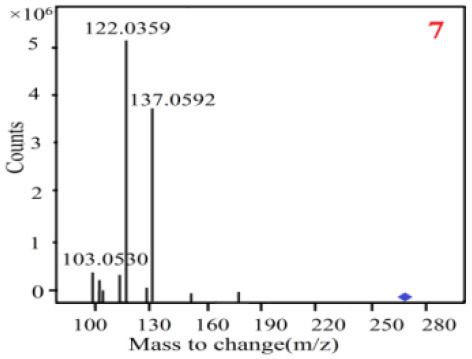	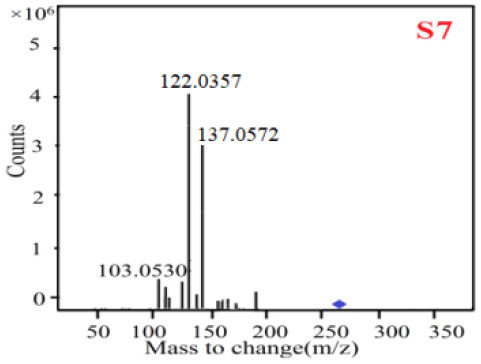
8	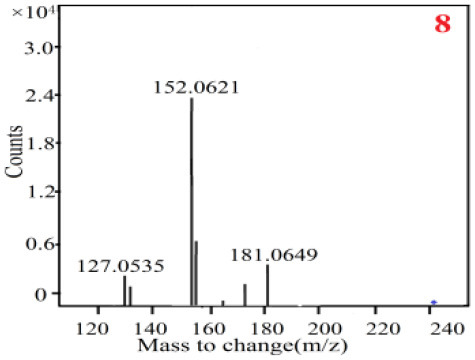	
9	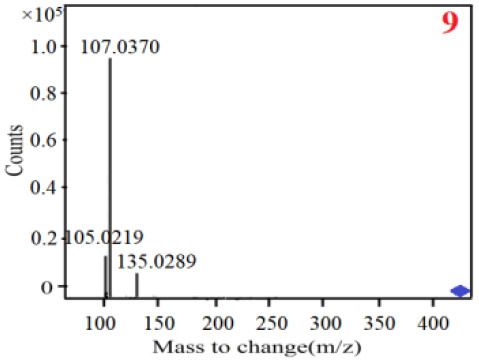	
10	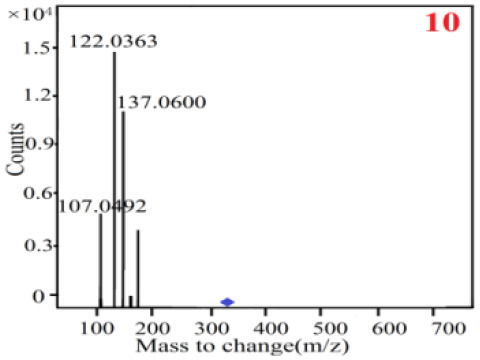	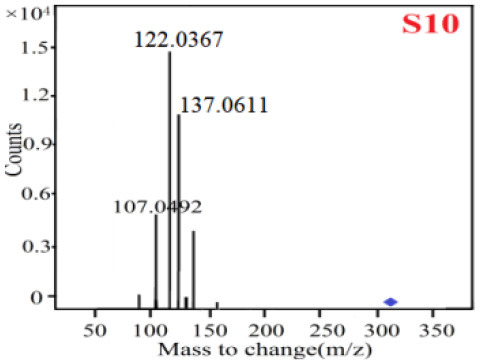
11	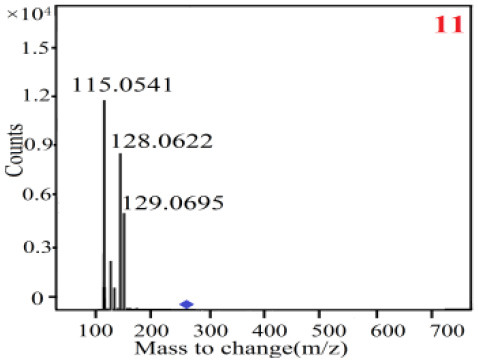	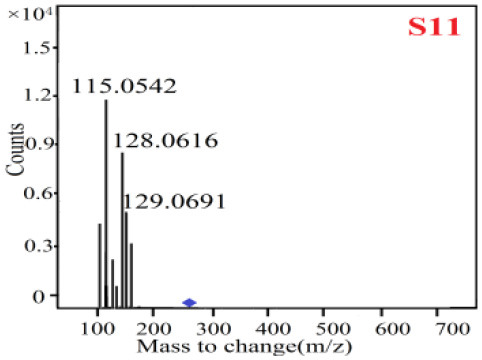

*The numbers 1–11 represent the standard compounds 1–11 in Figure 1*.

### Method Validation

#### Calibration Curves

The calibration curves of the 11 polyphenols were constructed using the peak areas from eight concentration levels as a function of the analyte concentrations. Good linearity was observed for each analyte (with the coefficient of determination [*R*^2^] >0.999 for each analyte) in this study (Table 4). The LOD and LOQ were within the ranges of 4–168 and 15–562 ng/ml, respectively.

**Table 4:** Calibration curves, linearity, limits of detection (LODs), and limits of quantification (LOQs) of the method used for assessing the 11 analytes.

**No**.	**Compounds**	**Calibration curve**	**R^2^**	**Linearity**	**LOD (ng)**	**LOQ (ng)**	**Precision**	**Stability**	**Repeatability**	**Average**
				**(ng)**			**RSD%**	**RSD%**	**RSD%**	**recovery (%)**
1	Schaftoside	Y = 0.52X – 8.68	0.999	5.53–548.64	0.31	1.03	0.64	0.68	0.77	97.69
2	Isoschaftoside	Y = 0.53X – 1.33	0.999	4.79–475.20	0.37	1.25	0.65	0.66	0.70	98.32
3	2,4,7-Trihydroxy-9,10-dihydrophenanthrene	Y = 0.39X + 1.02	0.999	2.93–237.42	1.12	0.38	0.39	0.31	0.41	91.25
4	Dihydroresveratrol	Y = 0.17X + 0.38	1	1.128–95.44	0.04	0.15	0.55	0.55	0.44	94.83
5	Coelonin	Y = 0.14X + 0.70	0.999	1.07–86.33	0.06	0.21	0.76	0.55	0.72	92.45
5	Lusianthridin	Y = 0.21X – 3.72	0.999	5.08–181.44	0.15	0.49	0.65	0.43	0.42	96.78
6	Nudol	Y = 0.14X + 0.03	1	0.93–75.09	0.16	0.53	0.51	0.50	0.44	90.65
7	Gigantol	Y = 0.63X – 0.05	1	8.84–877.39	0.27	0.91	0.28	0.16	0.20	92.55
8	4-Methoxy-2,5-phenanthrenediol	Y = 0.19X + 0.36	1	1.34–108.29	0.15	0.49	0.32	0.29	0.36	91.62
9	3-hydroxy-3,4,5-trimethoxybibenzy	Y = 0.13X + 0.07	1	0.96–77.99	0.07	0.24	0.73	0.19	0.37	93.46
10	Erianin	Y = 0.36X + 1.79	0.999	6.13–608.40	0.90	3.00	1.24	1.30	0.78	96.68
11	Tristin	Y = 0.85X – 4.26	1	12.26–1,216.80	1.68	5.62	0.79	0.77	0.98	99.17

#### Precision, Repeatability, Stability, and Recovery

Table 4 shows the precision data for the analytes from the working solution. The precision data had an RSD value < 1.5%. The RSD values for the analytes in the repeatability assay were <0.99%. The stability based on the analysis of the working solutions (with known intermediate concentration), which were kept at room temperature for 0, 2, 4, 6, 8, 12, and 24 h, also showed low *RSD* values. These results confirmed that the method we constructed had good precision, repeatability, and stability. The recovery experiments produced recovery rates in the range of 90.65–99.17%, with RSD values of <2% (Table 4). These further demonstrated the excellent accuracy of the established HPLC-DAD method and indicated that the variations in the quantification of the investigated polyphenols in the samples were highly acceptable.

### Analysis of the Polyphenols in Different *Dendrobium* Species Used for Preparing Shihu

The developed HPLC method was applied for quantitative analysis of the 11 investigated compounds in the four *Dendrobium* species used for preparing Shihu, and the results were summarized in Table 5. Four polyphenols, namely, compounds 1, 2, 4, and 11, were found in all the four *Dendrobium* species, and the contents of compounds 4 and 11 were very similar in the samples. This finding indicated chemical consistency among the four *Dendrobium* species. However, the samples also showed substantial variations for other compounds. For example, the content of compound 1 was 33.21 ± 9.34 mg/kg in *D. chrysotoxum*, about 8–10 times that in *D. huoshanense, D. nobile*, and *D. fimbriatum* (Table 5). The content of compound 2 was 67.89 ± 5.54 mg/kg in *D. nobile*, which was obviously higher than that in *D. huoshanense, D. chrysotoxum*, and *D. fimbriatum*. In addition, the total contents of the 11 investigated polyphenols were also quite different (Table 5). Compound 8, which has been reported in *D. nobile* (18), and compound 9, which has been reported in *D. nobile* (19) and *D. chrysotoxum* (20), were not detected in any of the four *Dendrobium* species in our experiments. Thus, the levels of 8 and 9 might be very low in the four *Dendrobium* species and may vary with the locality and harvest time of *D. nobile* and *D. chrysotoxum*.

**Table 5:** Quantification of 1–11 in the four *Dendrobium* species (mg/kg).

**Compounds**	***D. huoshanense***	***D. nobile***	***D. chrysotoxum***	***D. fimbriatum***
1	4.08 ± 0.98	4.06 ± 0.69	33.21 ± 9.34	2.87 ± 0.61
2	15.01 ± 2.26	67.89 ± 5.54	23.01 ± 2.31	5.15 ± 1.37
3	1.18 ± 0.29	–	0.09 ± 0.09	0.08 ± 0.08
4	1.82 ± 1.82	1.59 ± 1.59	0.97 ± 0.90	0.52 ± 0.27
5	–	–	–	0.09 ± 0.09
6	0.08 ± 0.08	0.68 ± 0.68	0.10 ± 0.10	–
7	0.93 ± 0.93	–	–	–
8	–	–	–	–
9	–	–	–	–
10	–	2.16 ± 2.16	–	–
11	4.10 ± 1.22	1.35 ± 1.02	0.78 ± 0.12	1.25 ± 0.84
Total	27.2 ± 7.58	77.73 ± 11.68	58.16 ± 12.85	9.96 ± 3.26

### Consistency Evaluations

#### Consistency Evaluation Based on Chemical Compositions

To evaluate the similarity of chemical compositions in the four *Dendrobium* species used for preparing Shihu, the correlation coefficients based on the investigated polyphenols in the four species were calculated by the cosine angle method and were used as similarity measures. As shown in Table 6, the correlation coefficients between the different species ranged from 0.299 to 0.906. *D. huoshanense* showed relatively higher similarity to *D. nobile* (correlation coefficient, 0.906) while similarities between *D. chrysotoxum* and the other three species were lower (correlation coefficients, 0.299–0.457). Based on the similarity evaluations of the investigated polyphenols, the chemical consistency of *D. huoshanense, D. nobile*, and *D. fimbriatum* was considered to be higher, making them appropriate for interchangeable use in the preparation of Shihu.

**Table 6:** Correlation coefficients based on the contents of compounds 1–11 in the four *Dendrobium* species used for preparing Shihu.

	***D. huoshanense***	***D. nobile***	***D. chrysotoxum***	***D. fimbriatum***
*D. huoshanense*	1	0.906	0.457	0.765
*D. nobile*		1	0.299	0.758
*D. chrysotoxum*			1	0.346
*D. fimbriatum*				1

*D. huoshanense, Dendrobium huoshanense; D. nobile, Dendrobium nobile; D. chrysotoxum, Dendrobium chrysotoxum; D. fimbriatum, Dendrobium fimbriatum*.

#### Consistency Evaluation Based on HPLC Fingerprints

**Figure 3:**
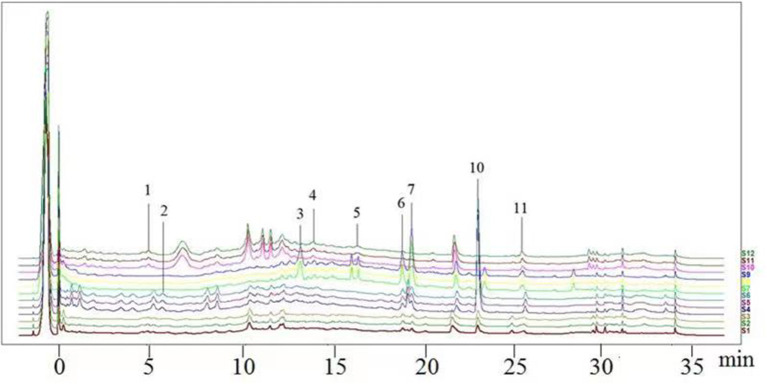
The overlapping HPLC characteristic chromatograms of the four *Dendrobium* samples. S1–S3: *D. chrysotoxum*; S4–S6: *D. fimbriatum*; S7–S9: *D. nobile*; S10–S12: *D. huoshanense*. *D. huoshanense, Dendrobium huoshanense; D. nobile, Dendrobium nobile; D. chrysotoxum, Dendrobium chrysotoxum*; *D. fimbriatum, Dendrobium fimbriatum*; HPLC, high-performance liquid chromatography.

The chromatographic fingerprint technique is a useful tool to control the quality of TCM formulations due to its outstanding advantages in the systematic characterization of the compositions of medicinal plants. In this study, the HPLC fingerprints of the four *Dendrobium* species (Figure 3) obtained by the developed HPLC-DAD method were analyzed using the Similarity Evaluation System of Chromatographic Fingerprints of TCM (the 2012 Edition) to evaluate the consistency of the four species used for preparing Shihu. The similarity analysis showed correlation coefficients of 0.615–0.916 between the species (Table 7), which were higher than the coefficients in analyses based on the 11 polyphenols. The correlation coefficients among *D. huoshanense, D. nobile*, and *D. fimbriatum* were all above 0.86, suggested their chemical compositions might be highly consistent. However, the correlation coefficients between *D. chrysotoxum* and the other three investigated species were only 0.575–0.716 (Table 7), which was remarkably lower than those for the other three species. These findings were consistent with the results of similarity analysis based on polyphenols and further suggested that *D. huoshanense, D. nobile*, and *D. fimbriatum* might be more appropriate for interchangeable use in the preparation of Shihu.

**Table 7:** Similarity evaluation based on the HPLC fingerprints of the four *Dendrobium* species.

	***D. huoshanense***	***D. nobile***	***D. fimbriatum***	***D. chrysotoxum***
*D. huoshanense*	1	0.916	0.898	0.716
*D. nobile*		1	0.864	0.660
*D. chrysotoxum*			1	0.575
*D. fimbriatum*				1

*D. huoshanense, Dendrobium huoshanense; D. nobile, Dendrobium nobile; D. chrysotoxum, Dendrobium chrysotoxum; D. fimbriatum, Dendrobium fimbriatum; HPLC, high-performance liquid chromatography*.

The interchangeable use of multiple plants to prepare a formulation has been recorded in many TCM books, such as *ChP*, and is widely employed in clinical practice. The 2015 edition of the *ChP* has included a total of 618 multi-origin TCM formulations (constituting 24.6% of the TCM formulations included in the ChP), of which 21 had more than four interchangeable plants. Shihu is a famous TCM formulation, and more than 50 *Dendrobium* species have been identified as the ingredients in Shihu according to the latest edition of the ChP. However, this practice would require the component herbs to share similar compositions, at least in terms of the bioactive agents, to ensure they can replace each other in drug preparation. Our study highlighted the need for more scientific and systematic studies to evaluate the consistency of such multi-origin TCM formulations.

## Conclusions

An accurate and rapid HPLC-DAD method was developed for simultaneous analysis of 11 phenolic compounds in the methanol extracts of the main original plants of Shihu. The quantification results obtained with the established method showed substantial variations in the polyphenol content in samples of different *Dendrobium* samples. Analyses of the consistency of the four *Dendrobium* species on the basis of the 11 investigated polyphenols and the HPLC fingerprints indicated that *D. huoshanense, D. Nobile*, and *D. fimbriatum* showed greater similarity and were more appropriate for interchangeable use in preparing medicines, while *D. chrysotoxum* showed rather a low similarity to the other three species (with correlation coefficients of 0.299–0.457 in similarity analysis based on polyphenols and 0.575–0.716 in consistency evaluation based on the HPLC fingerprints). More evidence is required to clarify the rationality of the interchangeable use of these four *Dendrobium* species in preparing the same medicine.

## Data Availability Statement

The original contributions presented in the study are included in the article/supplementary materials, further inquiries can be directed to the corresponding author.

## Author Contributions

A-LZ and J-WH finished all the experiments. LL helped with the data. QW provided some idea of the manuscript. N-DC provided the majority of the idea for the manuscript. G-LW helped to perform the UPLC-TOF-MSMS experiment. X-QL and H-MX helped to collect the plant material. W-HY helped with the similarity evaluation. All authors contributed to the article and approved the submitted version.

## Funding

This research was supported by the Major Science and Technology Projects of Anhui Province (201903a07020017), the National Natural Science Foundation of China (81573536 and 81274021), School Level Projects of West Anhui University (WXZR202029), Anhui Provincial Natural Science Foundation (1808085MH307), and Provincial Level Nature Science Foundation of Anhui Education Department (KJ2019A0628, KJ2018A0421, and KJ2016A747).

## Conflict of Interest

The authors declare that the research was conducted in the absence of any commercial or financial relationships that could be construed as a potential conflict of interest.

## Publisher's Note

All claims expressed in this article are solely those of the authors and do not necessarily represent those of their affiliated organizations, or those of the publisher, the editors and the reviewers. Any product that may be evaluated in this article, or claim that may be made by its manufacturer, is not guaranteed or endorsed by the publisher.
